# Community detection and management of mild cognitive impairment in Shanghai: a mixed-methods study

**DOI:** 10.1093/heapol/czaf025

**Published:** 2025-04-24

**Authors:** Yuan Lu, Dehua Yu, Yvonne Wells, Chaojie Liu

**Affiliations:** Department of Gerontology, Yangpu Hospital, Tongji University School of Medicine, 450 Tengyue Road,Tongji University Affiliated Yangpu Hospital, Shanghai 200090, China; Shanghai General Practice and Community Health Development Research Center, Tongji University School of Medicine, 450 Tengyue Road, Tongji University Affiliated Yangpu Hospital, Shanghai 200090, China; Lincoln Centre for Research on Ageing, La Trobe University, 1 Kingsbury Dr, Melbourne, VIC 3086, Australia; School of Psychology and Public Health, La Trobe University, 1 Kingsbury Dr, Melbourne, VIC 3086, Australia

**Keywords:** mild cognitive impairment, mixed-methods study, chronic care model, primary care

## Abstract

Dementia has been regarded as a priority in public health for healthy ageing. Mild cognitive impairment (MCI) detection and management is one of the strategies to confront the challenge of increasing burden of dementia. However, MCI is not well recognized or managed in primary care. This study aimed to assess system barriers relating to MCI detection and management in the community. A mixed-methods study was undertaken over the period from October 2020 to October 2022. First, a focus group study (*n* = 124) in Shanghai explored the experiences of general practitioners (GPs), people with MCI and their informal caregivers, and community health managers using thematic analysis. This was followed by 2 rounds of national Delphi surveys among 22 eligible experts to solicit their consensus on the system conditions needed for community detection and management of MCI. A questionnaire survey based on the Delphi consultations was conducted with GPs (*n* = 1253) recruited from 56 community health centres (CHCs) in Shanghai to quantify their knowledge, attitudes, and practice (KAP) toward community detection and management of MCI and perceived system barriers. The results were mapped and triangulated in line with the chronic care model (CCM) and the health system building blocks articulated by the World Health Organization. Potential system barriers were identified from eight themes: (i) lack of self-management skills and enablement; (ii) lack of family support; (iii) lack of community support; (iv) unprepared healthcare system; (v) health service delivery deterrence; (vi) inadequate clinical decision support; (vii) lack of case management; and (viii) misaligned clinical information systems. The primary care system in Shanghai is not adequately equipped to handle the task of detecting and managing MCI. Both intrinsic and extrinsic obstacles impede the successful conversion of MCI knowledge into desired actions. A systems approach is needed to confront the challenge of MCI detection and management in China.

Key messages

- Key stakeholders—including primary care providers, health services managers, and consumers—have not been adequately prepared or coordinated to address the challenges of detecting and managing mild cognitive impairments (MCI) in China.
- This mixed-methods study identified barriers to MCI detection and management in the community at the individual, organizational, and system levels in Shanghai, China. Our findings highlight gaps such as limited self-management skills and enablement, insufficient family and community support, an unprepared healthcare system, deterrents to health service delivery, inadequate clinical decision support, ineffective case management, and misaligned clinical information systems.
- Addressing these barriers is crucial to fostering continuous, productive interactions between care providers and care recipients, ultimately improving MCI management and outcomes.

## Introduction

Dementia has been recognized as a priority in global public health (WHO 2021). Dementia is characterized by progressive cognitive decline, which leads to dependency among affected people, causing an overwhelming burden to family caregivers and society (Jia et al. 2020b). It has been reported that medications for dementia are relatively expensive, not always effective, and rarely covered by health insurance (Wang et al. 2019). Mild cognitive impairment (MCI) is a transitional cognitive stage that lies between normal aging and dementia (Petersen 2016). Approximately 45% of dementia cases could be prevented or delayed if timely interventions were effectively implemented (Livingston et al. 2024). Therefore, efforts have focused on detecting and managing people with MCI as one of the strategies to confront the challenge of the increasing burden of dementia (Owens et al. 2020).

Currently, MCI interventions are mainly focused on the management of modifiable risk factors and non-pharmacological cognitive interventions in the community (Jia et al. 2020a). Multicomponent interventions combining cognitive training and physical exercise have proven to be the most effective approach to improving cognition function for people with MCI (Gavelin et al. 2021). A primary care-dominated health system has been proven to be the most cost-effective for ensuring population health (Potter et al. 2023). Most community-based MCI programs aim at early diagnosis and prevention of dementia (Global Council On Brain Health 2022). According to the current evidence on detecting and managing MCI, general practitioners (GPs) are situated in a prime position to assist patients in managing MCI (Owens et al. 2020). In a primary-care-dominated health system, GPs serve as the initial point of contact for patients and take charge of delivering crucial medical services to the community (Ventres et al. 2024). In China, GPs have a responsibility within the primary care system to oversee the coordination of care and liaise with other care providers on behalf of their patients (Xiong et al. 2023).

However, MCI is not well recognized or managed within primary care settings, both in China and other countries (Sabbagh et al. 2020, Jia et al. 2020a). Previous evidence from both quantitative and qualitative studies has highlighted that the main stakeholders of MCI detection and management have not been adequately prepared to confront the current situation (Poppe et al. 2020, Zhai et al. 2024). For example, one meta-analysis indicates that more than half of MCI cases have not been identified by GPs, and only 10.9% of those cases are documented in medical records, which raises concerns about GPs’ limited knowledge, attention, and actions on MCI (Jia et al. 2020b). Studies have found numerous barriers to the public and even GPs acknowledging the value of MCI detection and management and the lack of system resources to act (Lu et al. 2022b, Borson et al. 2023).

Extensive studies have been undertaken on MCI screening, diagnosis, and treatment strategies targeting different stakeholders (Global Council On Brain Health 2022). For example, the Cognitive Training Support Program (CTSP) provides counselling services to enhance the engagement and adherence of elderly individuals with MCI to the cognitive training intervention through an online group meeting format (de Jager Loots et al. 2024), while the Isupport program delivers online support to informal caregivers for people with MCI (Xiao et al. 2021). In Australia, a nationwide dementia-focused continuing medical education program (including MCI) is offered to medical workers (Casey et al. 2020). Interactions between care recipients and care providers occur within a system context that includes factors such as infrastructure facilities, public health policies, and the organizational culture (Airhihenbuwa et al. 2021). However, few studies have elaborated on the complexity of MCI detection and management from a systems perspective (Liss et al. 2021).

There is a notable lack of literature that details the experience of people living with MCI and the challenges they face in seeking medical care and support services. Therefore, this study was designed to address the gap in the literature by assessing system barriers relating to community detection and management of MCI in Shanghai, China, using a mixed-methods approach involving multiple stakeholders. Despite being the backbone of the primary care workforce, GPs are not assigned a gatekeeping role in the Chinese health system (Huang et al. 2024). Through a sequential exploratory design, qualitative and quantitative results were integrated using a systems lens, with GPs, an emerging health professional in China (Chen et al. 2022), being placed in the centre for MCI detection and management in the community.

## Methods

### Study setting

Community detection and management of MCI in primary care is a daunting task in China. A recent meta-analysis indicated that the prevalence of MCI in China among people over 55 years old in the community is 12.2% (Lu et al. 2021). In 1997, the Chinese government decided to strengthen the primary care sector through GP-led community health services (National Health Commission 2000). However, there has been a shortage of GP workforce, and hospital domination remains a prominent feature of the Chinese healthcare system (Chen et al. 2022). In 2022, China had only 4.5 million registered/assistant GPs (3.4 per 10 000 population), far behind the governmental goal of 5 GPs per 10 000 population by 2030 (National Health Commission 2020a). Many GPs, particularly assistant GPs, transitioned from medical workers without a formal university degree, and MCI-related training is lacking (Lian et al. 2019). The shortage of community nurses in China is even more serious than that of GPs (Zhan et al. 2019). While the vast majority of GPs are employed by community health centres (CHCs), community and institutional aged care and support facilities are funded and overseen by a completely separate governmental portfolio.

This study was carried out in Shanghai, which is the first city in China to have reached the threshold of an ageing society (Chen and Chen 2006). China adopts a community-oriented approach to primary care (Xu and Chow 2011). Unfortunately, community detection and management of MCI has not been regarded as a priority in primary care in China. Under such circumstances, in 2018, the Shanghai government initiated the ‘Friendly Community Program’ for older people with cognitive impairment (Shanghai Civil Affairs Bureau 2020). This program aims to not only guarantee the safety and welfare of people living with cognitive impairment, but also to encourage and enable every member of the community to engage in supporting people with cognitive disorders. The program requires CHCs and GPs to help informal family caregivers better care for those with MCI, while local governments and community organizations offer assistance with activities of daily living.

CHCs are committed to providing essential public health services (EPHS) to all (The State Council 2012). The national EPHS package in 2022 suggests the inclusion of cognition assessment in preventive physical examination services (Lu et al. 2022b). However, top-down enforcement to establish MCI programs in primary care has not been accompanied by an additional governmental budget. Social health insurance programs have also been slow to adjust to the changing scope of services (Lee et al. 2022). MCI-related services, if available in CHCs, are often offered for free under the ‘Friendly Community Program’, despite the already scarce resources (Shanghai Civil Affairs Bureau 2020).

Community detection and management of MCI involves health professionals within and outside the primary care sector as clinical guidelines require specialist confirmation of MCI diagnosis (Jia et al. 2020b). Although GPs in China are to lead a multidisciplinary team in CHCs for managing chronic conditions (Dai et al. 2021), there is no compulsory ‘gate-keeping’ role for GPs to prevent patients from bypassing primary care (You et al. 2021). Instead, a voluntary primary care contracting system is implemented in an effort to maintain continuity of primary care (The State Council 2016). Community residents are encouraged to enter a contract, free of charge, with their local GPs (or GP-led teams) in CHCs to access certain benefits, such as prioritized medical appointments and higher social insurance subsidies. However, those with a contract still have the freedom to bypass primary care when seeking specialist care in hospitals.

### Ethics approval

Ethical approval was granted by the authors’ institutions. Written informed consent was obtained before the commencement of the focus group study. Implied informed consent was obtained from each participant in the Delphi expert consultation and the online anonymous survey of GPs. All participants were provided with an introduction to the study before data collection and had the right to withdraw at any time without providing an explanation.

### Study design

This project adopted a mixed methods design (Vedel et al. 2019), signifying the most comprehensive integration of two more study designs to explore relatively new and unexplored areas. A sequential exploratory approach was used, starting with a qualitative study to explore stakeholder experiences relating to MCI, followed by quantitative studies to examine what GPs did in community detection and management of MCI. The design of both studies was guided by the chronic care model (CCM) (Davy et al. 2015), which is an organizational approach that emphasizes continuous productive interactions between care providers and care recipients. The CCM aligns well with the principles of primary care: community participation, intersectoral coordination, appropriate technologies, and support mechanisms (Ventres et al. 2024). It underscores the significance of prepared, proactive practice teams and well-informed, motivated patients, along with a system platform that facilitates effective communication between them (Davy et al. 2015). The CCM has been proven to be effective in managing chronic conditions such as hypertension, diabetes, osteoporosis, and asthma (Reynolds et al. 2018).

These studies answered three interconnected research questions:

(i) What are the experiences of people living with MCI and how are they supported by informal and formal care providers?—These questions would be best answered through a qualitative study design, because it enables investigation into the everyday experiences of people by setting aside the researchers’ preconceived assumptions (De Haan et al. 2021). In this project, we organized focus groups for people living with MCI, GPs with experience in managing MCI, and CHS managers involved in MCI programs, respectively. The separated arrangements minimized power imbalance and ensured openness in communications (Wilkinson 1998).

(ii) What do GPs or CHCs need to do in managing MCI?—With the absence of practice guidelines, a Delphi expert consultation study was conducted to answer this question (Niederberger and Spranger 2020). It served two purposes: first, the stakeholder experiences revealed in the focus group studies could be mapped against the Delphi results (expert consensus) in order to assess the performance of the existing MCI management practices; second, a quantitative survey tool could be developed to investigate the preparedness of GPs and CHCs in detecting and managing MCI.

(iii) What are the gaps and challenges in implementing the expert consensus on managing MCI?—A quantitative survey was conducted to investigate the individual, organizational, and system conditions required for GPs in community detection and management of MCI.

## Data collection and analysis

**Figure 1.**
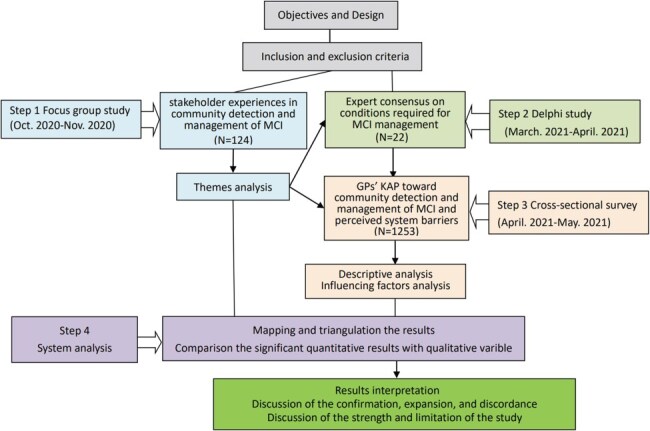
Study process diagram.

This project involved four steps (Fig. 1).

### Step 1:focus group study

Given that a small proportion of GPs had experience in MCI management, a purposive sampling strategy was employed to choose participants from six CHCs in Shanghai that offered an MCI program, ensuring a balanced representative of age, gender, and MCI-related experience across the three categories of participants: people with MCI and their informal caregiver; GPs; CHC managers. All of the focus groups were conducted face-to-face at the participating CHCs with the coordination of the first author. Although the focus group sessions were guided by a list of question prompts in line with the CCM (Lu et al. 2022c), participants were encouraged to freely express their views. Data were digitally recorded, transcribed, reviewed, and analysed after each focus group session to inform the need for further data collection. The final sample size was determined by information saturation (Hennink et al. 2019). This resulted in a final sample of 42 GPs (6 sessions), 32 people with MCI and 32 informal caregivers (4 sessions), and 18 CHC managers (3 sessions).

Content analyses were conducted utilizing a blend of deduction (guided by the CCM framework) and induction (open codes) coding approaches (Lu et al. 2022c). Common themes were extracted and verified by examining the experiences of all stakeholders.

### Step 2: Delphi consultation study

The research team explored a consultation checklist containing 44 items measuring MCI-related aspects from the provider, patient, and environmental perspectives based on the results of the focus group study and the existing literature (Rosenstock et al. 1988, WHO 2010, Michie et al. 2011). A convenience sample of experts (*n* = 24), covering the expertize of general practice, neuropsychology, public health, and community health service management, was recruited across China. Participants were instructed to evaluate the relevance and significance of each item using a five-point Likert scale and to suggest necessary modifications. The checklist was revised by incorporating the expert feedback, before a repeat consultation was conducted with the same participants. In our study, expert consensus (as indicated by more than 80% agreement with a rating of ‘important’ or ‘essential’) was achieved through two rounds of consultations, with 22 experts completing both rounds of consultations. Over 91.6% of these experts agreed that 47 necessary conditions are required to enable effective primary care responses to community detection and management of MCI in China (Lu et al. 2022d).

### Step 3: cross-sectional survey

GPs recruited from 56 (22.7%) CHCs in Shanghai completed an anonymous online questionnaire survey. The survey tool was derived from the Delphi consultation in addition to support from the existing literature (Hogg et al. 2008, WHO 2008, 2010, Michie et al. 2011). Those who volunteered to participate in the survey were asked to rate their knowledge (8 items), attitudes (13 items), and practice (11 items) toward community detection and management of MCI, as well as challenges relating to patient engagement (14 items), work conditions (12 items), and environmental supports (7 items) (Lu et al. 2022a). A total of 1253 GPs returned valid responses. A summed score, ranging from 0 to 100, was calculated for each of the domains: a higher score indicates a higher level of knowledge, more favourable attitudes, higher intentions to follow practice guidelines, and higher perceived barriers in patient engagement, work conditions, and environmental support. Student *t*-tests, ANOVA, multivariable linear regression, and structural equation modelling were conducted to determine the individual, organizational, and system factors associated with the KAP of GPs (Lu et al. 2022a, 2022b).

### Step 4: system analysis

The qualitative and quantitative findings were integrated using the CCM framework to provide an overall picture of how the primary care sector in Shanghai responded to the challenges of community detection and management of MCI. The analysis of integration fit (Meta-inferences) resulted in three possible outcomes: confirmation, which happened when the results from both types of data aligned with each other; expansion, which occurred when the results addressed distinct aspects of a single phenomenon or described complementary facets of the main phenomenon of interest; and discordance, which arose if the qualitative and quantitative findings contradicted each other (Liu et al. 2021).

The study results were presented according to the Mixed Methods Article Reporting Standards (MMARS) (Levitt et al. 2018). The integrated results were summarized in line with the eight elements embedded in the CCM (Davy et al. 2015): (i) Self-management, which refers to the support that motivates an informed and empowered patient to engage in collaborative goal setting, problem-solving, and continuous adjustment and improvement of intervention measures (Allegrante et al. 2019). (ii) Family support, which comes from biologically, legally, or emotionally attached family members, who are the most valuable asset for people living with chronic conditions, in particular those with functional limitations in their daily lives (Rosland and Piette 2010). (iii) Community support, which plays two possible roles: mobilization of communities to create healthy environments and various forms of advocacy (Simonsen et al. 2021). (iv) Health system arrangements, which consist of various elements of health systems, grounded in the six WHO health system building blocks: health workforce, service delivery, access to essential medicines, health information systems, financing, and leadership/governance (WHO 2010). (v) Health services delivery, which is an approach that ensures coordinated care and support at the appropriate time by establishing connections among all individuals in the care delivery process (Lennox-Chhugani 2021). (vi) Clinical decision support, which includes support for screening, diagnostic testing, and treatment options (Thomas et al. 2021). (vii) Case management, which is defined as a collaborative process where a case manager orchestrates efforts across various organizations to achieve high-quality, cost-effective outcomes (Campagna et al. 2024). (viii) Clinical information systems, which are critical for the effective management of chronic diseases by tracking, monitoring, and providing feedback on interventions and outcomes (Siminerio 2010, Davy et al. 2015).

**Figure 2.**
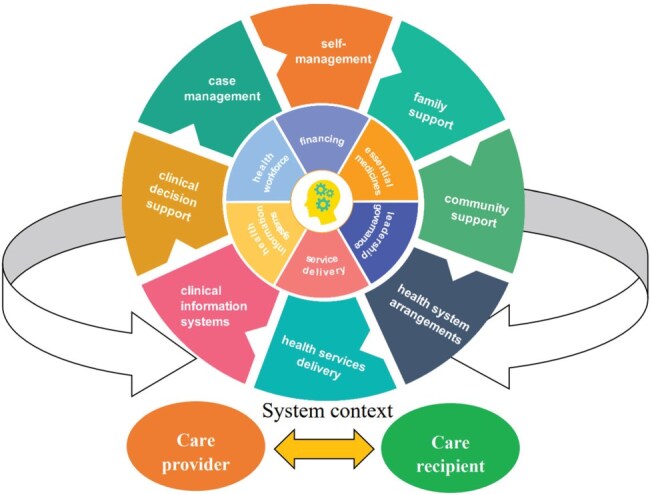
Theoretical framework for community detection and management of MCI.

Further evidence about the system challenges was sought from additional policy documents and government records, such as the Age Friendly Society policy (National Health Commission 2020b), MCI training for GPs (Shanghai Municipal People’s Government 2024), MCI components in essential public health packages (National Health Commission 2022c), and health insurance coverage of MCI-related service claims (Shanghai Civil Affairs Bureau 2020). It is important to note that system challenges relating to each CCM element always involve multiple system blocks, and one challenge might be applicable to multiple CCM elements (Fig. 2).

Data analyses were conducted utilizing NVivo V.10 (QSR International, 2012), IBM SPSS software version 27.0, and SmartPLS 3.3.3.

## Results

Table 1 presents the integrated results of this project. Potential system barriers were identified from eight themes.

**Table 1.** Meta-inferences of qualitative and quantitative evidence to support the expert consensus on conditions required for effective community detection and management of MCI

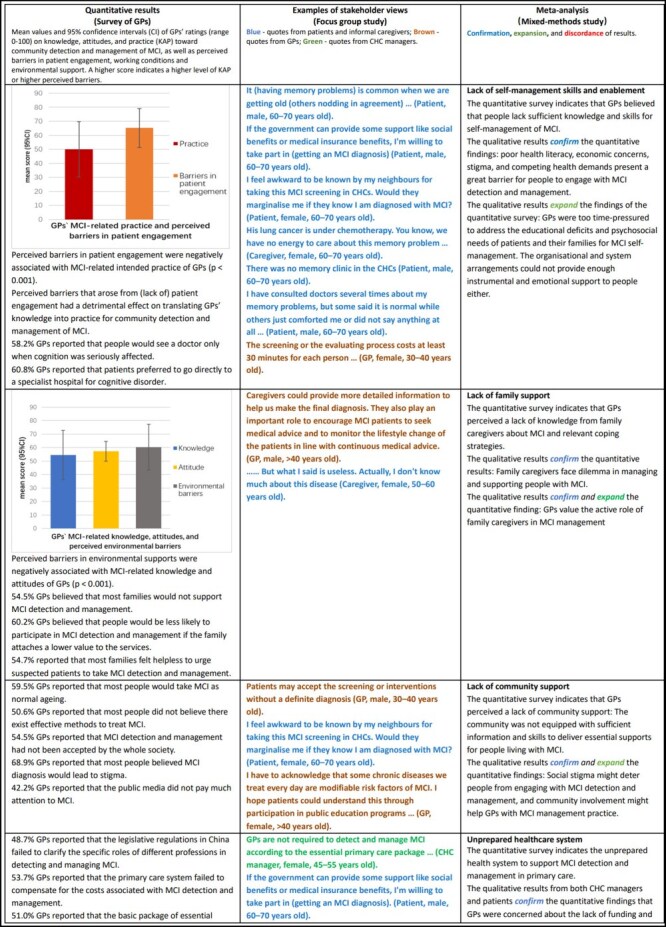
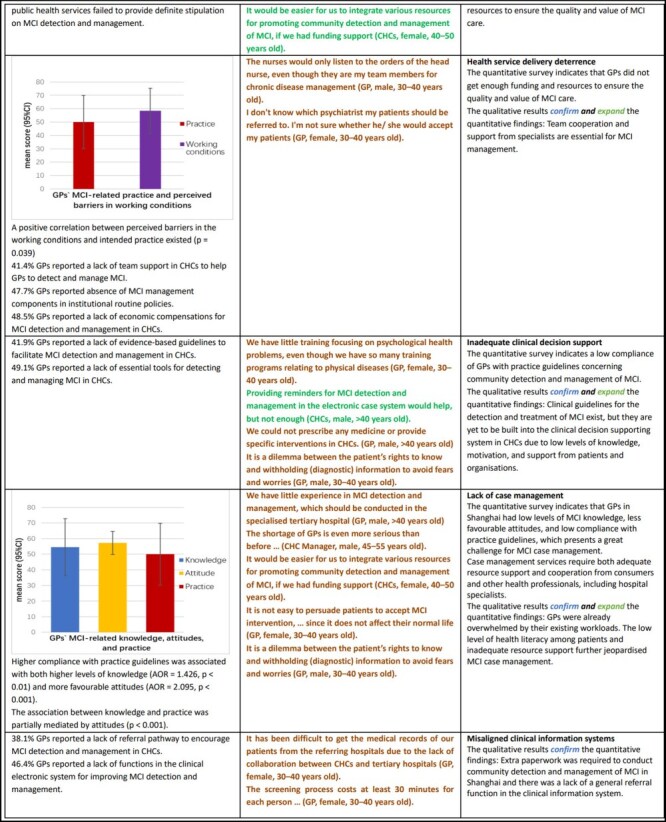

### Self-management

There was a lack of self-management skills and enablement among those living with MCI. GPs’ intended practice related to MCI was negatively correlated with perceived barriers in patient engagement (*P* < .001), which had a negative impact on converting GPs’ knowledge into practice for identifying and managing MCI in the community (*P* < .001). The structural equation model confirmed indirect effects of knowledge on intended practice via attitudes (84.6%) and perceived extrinsic barriers (−15.4%).

### Family support

Family was the major source of support for those living with MCI despite the limited understanding and guidance provided by family members. Over half (54.5%) GPs believed that most families would not support detecting and managing MCI. Perceived barriers in the environmental supports were negatively linked to GPs’ knowledge and attitudes towards MCI (*P* < .001).

### Community support

MCI was often perceived as a normal ageing process: 59.5% GPs believed most people held this view. Therefore, 54.5% GPs did not believe MCI interventions would be accepted by the society. On the contrary, 68.9% GPs held the view that people believe MCI diagnosis is linked to social stigma.

### Health system arrangements

There was a consensus among all stakeholders that the current health system was not readily prepared to implement community detection and management of GPs. This was echoed by the views of GPs in terms of inadequacy of legislative regulations (48.7%), payment arrangements (53.7%), and the essential public health program (EPHP) policy (51.0%).

### Health services delivery

The lack of individual, organizational, and system support led to the inaction of CHCs and a lack of professional preparedness of GPs to take on the task of community detection and management of MCI. While 41.4% GPs reported an absence of team support, 47.7% reported a shortage of managerial support. There was a positive relationship between intended practice and perceived barriers in the working conditions (*P* = .039).

### Clinical decision support

Although China issued clinical guidelines for screening, diagnosis, and management of MCI, 41.9% GPs reported having no knowledge of evidence-based guidelines of MCI management for CHCs. In addition, 49.1% GPs reported a lack of essential tools, and 38.1% felt a lack of referral pathways to obtain specialist support.

### Case management

Low knowledge, unfavourable attitudes, and low intended compliance with practice guidelines were common in GPs for community detection and management of MCI. Attitudes partially acted as an intermediary between knowledge and practice (*P* < .001). There was a lack of motivation and environmental support for GPs to engage in MCI case management. GPs were already overwhelmed by their existing workloads. The low level of health literacy among patients and inadequate resource support further jeopardized MCI case management.

### Clinical information systems

There was a lack of decision-support functions to guide practice through education, reminders, and other decision-support interventions, such as flow sheets, performance standards, access to specialists for consultation, and checklists for patient visits. This was confirmed by 46.4% GPs who reported a lack of required functions in their clinic electronic systems.

## Discussion

This mixed-methods study identified system barriers in MCI detection and management in Shanghai, China. GPs had neither adequate knowledge about nor favourable attitudes towards community detection and management of MCI. They also perceived barriers from patient engagement, their working conditions, and the environmental supports. Perceived extrinsic barriers hinder the conversion of knowledge into practice in detecting and managing MCI in the community.

In addition to the lack of professional competency, significant system barriers lie ahead to enable GPs in Shanghai to properly fulfill their assigned role as case managers for MCI. In North America and Europe, the majority of case managers in primary care are registered nurses (Hu et al. 2021). In China, however, GPs or teams led by GPs are supposed to play the case manager role (Zhan et al. 2019). This is partly because the shortage of nursing and allied health workforce is even more serious than that of physicians in the primary care sector in China, and partly because Chinese health professions are dominated by medicine and nurses are still grappling with defining their professional identity (Zhan et al. 2019). GPs are often deemed inferior to hospital specialists in China, and hospital specialists have limited, if any, incentives to support GPs with diagnosis and prescriptions (Yip et al. 2019).

There is robust evidence to indicate that self-management support is effective for improving chronic disease outcomes and applies especially well to preventive interventions involving lifestyle modification (Allegrante et al. 2019, Barnes et al. 2023). However, patients’ lack of self-management skills and family support was a perceived barrier according to the findings of this project. Primary care professionals can play an important role in educating patients and motivating them to engage in self-management of chronic diseases, including MCI, but health workers in many health systems, including in China, have been unable to adequately address patients’ and families’ educational and psychosocial needs due to time constraints (Rochfort et al. 2018). Shrinking family size, increasing population mobility, and declining co-residence present a significant challenge for family to support patients with MCI in China (Feng et al. 2020). These findings are consistent with the results of this project.

The low levels of organizational and system support were also linked to GPs’ low compliance with practice guidelines according to the findings of this project. Managing chronic conditions like MCI effectively necessitates substantial alterations to the current health system arrangements to accommodate the provision of chronic care (Bodenheimer et al. 2002). China has just started to promote community detection and management of MCI (Shanghai Civil Affairs Bureau 2020). Without a robust referral mechanism, the organizational boundaries make the referral of patients to hospital specialists challenging, prompting trials of developing organizational networks (Yu et al. 2017). Unlike in some other countries, such as Australia (Rahman et al. 2019), where GPs can refer their patients for a comprehensive assessment from an Aged Care Assessment Team (ACAT), such assessment services are absent from China. Clinical guidelines for detecting and treating MCI exist, but they are yet to be built into the clinical decision-supporting system (Dai et al. 2019). Furthermore, most clinical information systems in China are owned by individual health organizations and often lack interoperability (Yip et al. 2019).

The results of this study carry important implications for the development and execution of programs aimed at detecting and managing MCI in China’s community. A systems approach is needed to tackle the vicious circle of interconnected barriers resulting from unprepared providers, hesitant patients, and misaligned environments. All of the aforementioned issues are interconnected and must be addressed collectively. Efforts must be exerted across all of the WHO’s health system building blocks (WHO 2002).

### Building a solid and competent primary care workforce

Primary care provides most services needed by people living with chronic conditions (Rothman and Wagner 2003). GPs are usually assigned the responsibility of developing care plans for people with chronic conditions, providing relevant medical treatment, assessing intervention outcomes, and adjusting the plan accordingly (Khanassov and VEDEL 2016). The care plan forms a foundation for case management. Training needs to be strengthened to address the knowledge and skill gaps of GPs in detecting and managing MCI in China’s community. However, knowledge and skill gains are not enough, as they are associated with high levels of perceived external barriers (Lu et al. 2022b). Educational interventions for GPs need to foster the attitudes and behaviours required to address MCI. While extrinsic barriers could deter the efforts of some intrinsically motivated GPs, a systems approach is necessary to mitigate external obstacles to best practice, encompassing but not limited to aligning policy objectives, securing adequate funding, fostering coordination among various service levels, obtaining managerial support, and engaging in public education and community mobilization efforts.

### Enhancing patient engagement and participation

Primary care must be redirected towards supporting self-management, with an emphasis on hurdles in accommodating the increasing number of individuals with MCI. Family members are usually the first to notice the cognitive impairment of their loved ones, and to advise visits to healthcare providers (Jordan et al. 2020). Therefore, it is essential to facilitate family involvement in illness management and focus on family members’ unique roles. A systematic review has yielded moderate evidence indicating that early intervention can enhance caregivers’ well-being and capacity to provide care (Bayly et al. 2021). Isupport is a successful online program developed by the WHO, aimed at helping informal caregivers to enhance their knowledge and skills related to dementia. In Australia, various stakeholders believe the adaptation of the WHO ISupport would strengthen informal caregiver education and optimize support for them (Xiao et al. 2021). An adapted version of Isupport tailored to the needs of MCI can be developed in Shanghai based on the current ‘Friendly Community Program’ for older people with cognitive impairment.

### Fostering a supportive work culture

An organization’s culture and overall commitment to quality improvement of disease could influence GPs’ behaviour. The healthcare organization has a responsibility to unite healthcare personnel, define the roles of healthcare teamwork, equipping them with the necessary expertize and tools, and connecting them to community resources for managing patients with chronic problems (Loizeau et al. 2021). The quality of care is determined by effective interaction between patients and healthcare professionals as well as by successful within-team cooperation among different healthcare professionals.

### Streamlining care processes with support from modern information technologies

As technology advances, decision support and information technology have become intertwined. Modern technology could be applied to cope with the complex process of MCI detection and management during GPs’ routine care (El Asmar et al. 2021). Modern information technologies can not only be used in supporting the clinical decision-making of care providers but also in enhancing the self-management of patients (Siminerio 2010, Davy et al. 2015). Telemedicine enables effective and efficient delivery of healthcare services beyond the traditional boundary of healthcare settings (Freed et al. 2018). An ideal clinical information system for primary care follows patients rather than being confined within the boundary of primary care facilities. Previous studies indicate that the usefulness and compatibility of information systems may encourage healthcare professionals and patients to adopt a telemonitoring system for the management of chronic disease (Chatterjee et al. 2021).

### Aligning incentives with good practice

Proper funding and infrastructure are essential. CHC managers in our focus group study found it convenient to justify not providing infrastructure support for this function, citing policy ambiguity and a scarcity of funding sources as reasons (Lu et al. 2022c). Adding MCI-related services to the existing load of GPs without proper financial compensation is not sustainable and will hinder the scale-up of such services. In addition, without investment in facilities and human resources, it is difficult to ensure the quality of this work. While it is premature to determine whether reimbursement will result in long-term practice improvement, the project’s findings suggest that linking economic incentives to provider processes and patient outcomes holds promise.

### Mobilizing society

China has initiated a long-term health initiative known as Healthy China 2030 (Tan et al. 2017). Healthy China 2030 represents a significant advancement in ensuring the health of the Chinese population by promoting the participation of the entire society in the policy of ‘Health for All, and All for Health’. The ‘Friendly Community Program’ represents an attempt to adopt this policy (Shanghai Civil Affairs Bureau 2020). However, the fragmentation of activities can jeopardize the eventual outcomes (Yip et al. 2023). Better coordination between the community services overseen by the civil affairs authority and the primary care sector overseen by the health authority is needed, which usually requires a higher level of authority to facilitate.

This study used a sequential exploratory design with both qualitative and quantitative interpretation to bring together diverse information, aiming to comprehend the current status of the primary care sector in Shanghai in response to detecting and managing MCI in the community and the difficulties that it faces in doing so. The CCM framework was applied to enable the research through a systems lens (Davy et al. 2015). We examined all of the elements embedded in the CCM from the perspectives of consumers and providers. Shanghai is the first city in mainland China to have initiated a ‘Friendly Community Program’ for older individuals with cognitive impairment. Experiences and lessons can be learnt by other regions, although the Shanghai model cannot be directly copied due to great regional disparities in socioeconomic and health development in China (Meng et al. 2019). A systems approach is needed to handle the challenge of MCI detection and management in China.

Further research needs to be focused on testing the effectiveness of the CCM-based interventions on MCI detection and management, with an aim to address the limitations of this project. Moving forward through a systems approach requires a comprehensive mapping of all the CCM elements, including their intertwined links. The ‘Friendly Community Program’ provides an opportunity for Shanghai to develop innovative, collaborative mechanisms across sectors. Opportunities exist in the development of innovative approaches to measure the success of the CCM-based systematic intervention.

## Limitations

The project has several limitations. Firstly, this project was conducted in the primary care sector and hospitals were excluded, although hospital specialists play an important role in MCI-related services. Secondly, the study design is cross-sectional and observational. It prevents us from drawing causal conclusions. Thirdly, self-reported data were gathered, and we did not have access to real-world practice data; recall bias cannot be excluded. Finally, because the MCI screening initiative was still in its initial developmental stage in Shanghai, very few GPs had experience in detecting and managing MCI.

## Reflexivity statement

The authors include two females and two males. While two of the authors specialized in health policy in China, and the other two specialized MCI community management. Three authors have extensive experience conducting both qualitative and quantitative fieldwork in primary healthcare.
